# Supplementary material for: DMRforPairs: identifying Differentially Methylated Regions between unique samples using array based methylation profiles
Source: BMC Bioinformatics. 2014 May 15;15:141. doi: 10.1186/1471-2105-15-141 (PMC4046028; doi:10.1186/1471-2105-15-141)
Supplement: Additional file 2 — DMRforPairs output for the comparison of A431-MCF7 and NA17018-NA17105. Please start from the HTML files in each folder. Available via the BMC Bioinformatics website. [file 1471-2105-15-141-S2.zip › 1394847754114233_MOESM2_ESM/A431_MCF7/figures/22638.pdf]

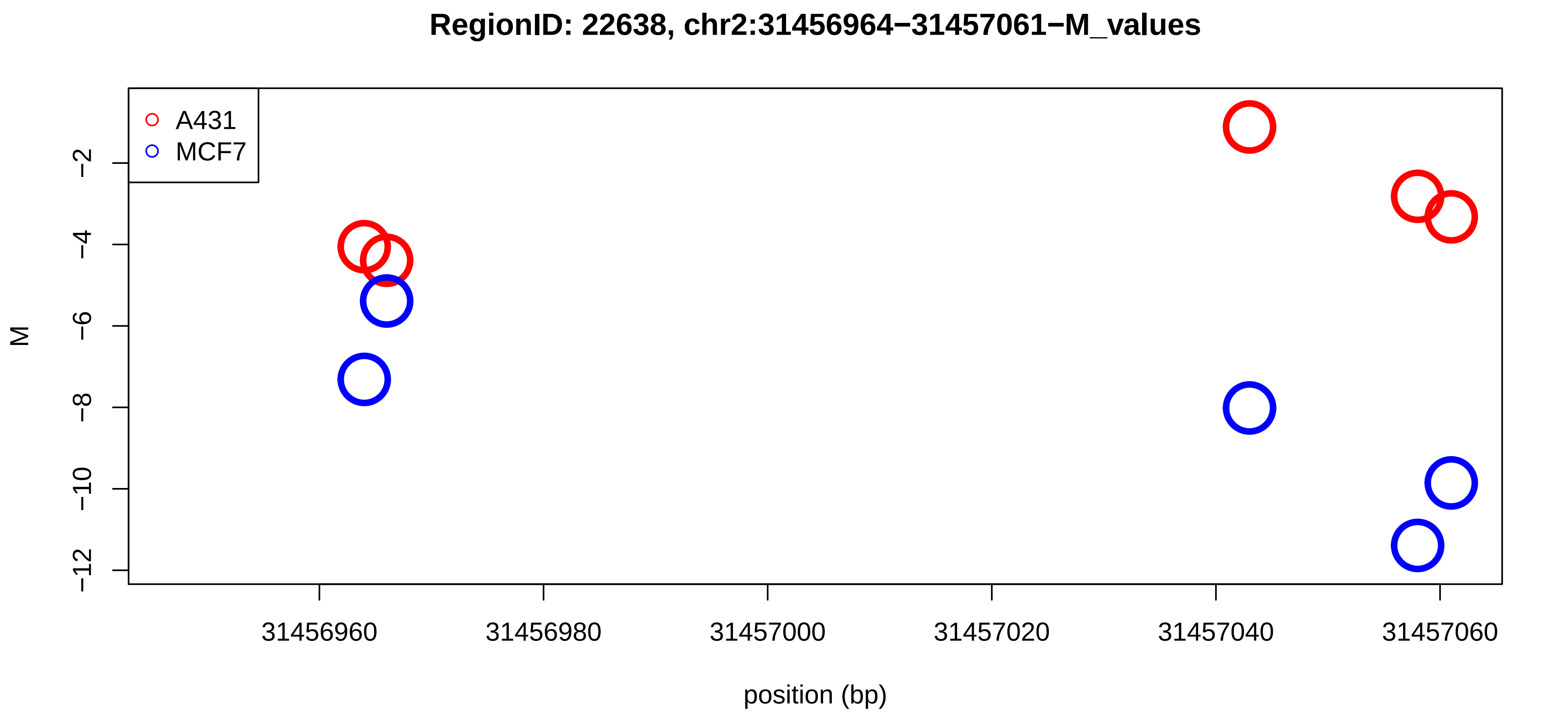
RegionID: 22638, chr2:31456964−31457061−M_values
A431
MCF7
M
−2
−4
−6
−8
−10
−12
31456960
31456980
31457000
31457020
31457040
31457060
position (bp)

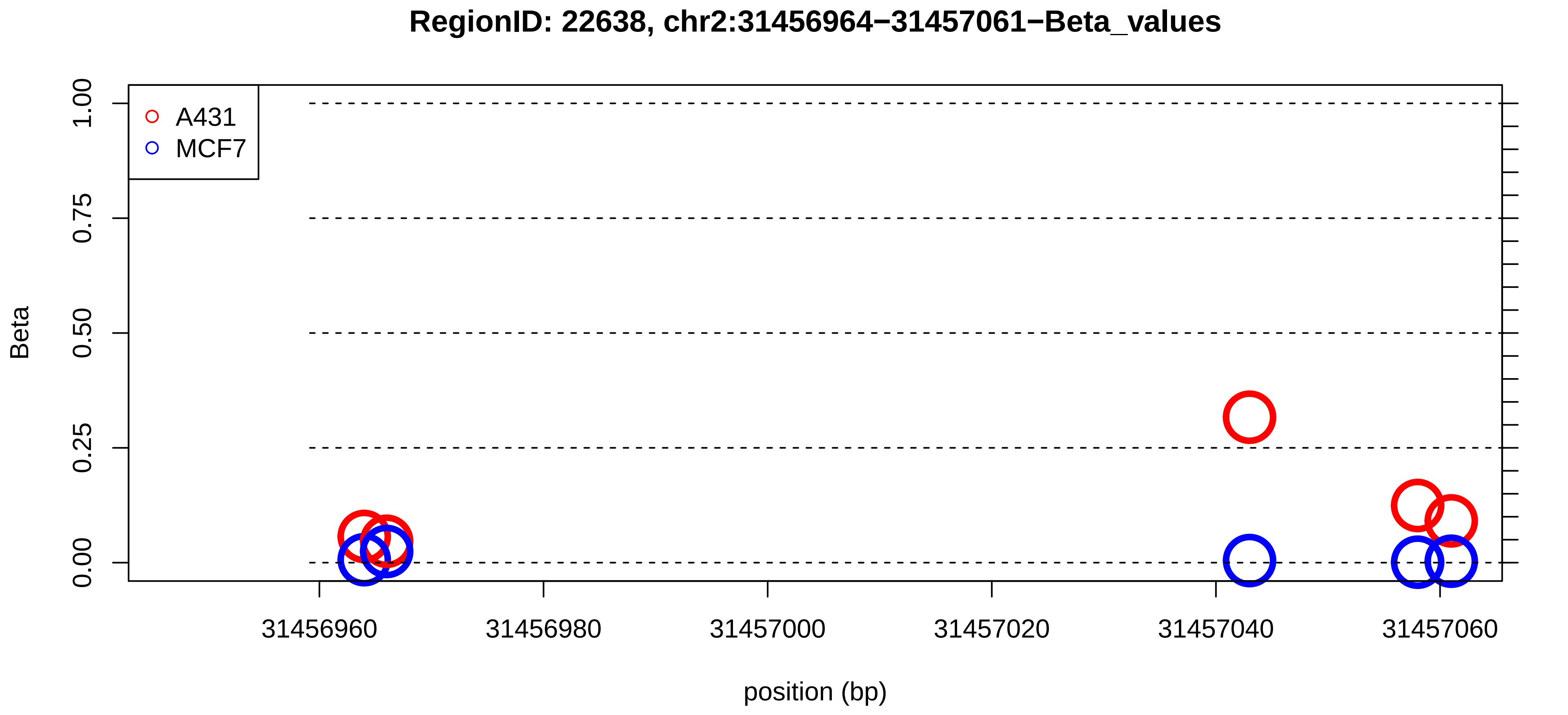
RegionID: 22638, chr2:31456964−31457061−Beta_values
A431
MCF7
Beta
1.00
0.75
0.50
0.25
0.00
31456960
31456980
31457000
31457020
31457040
31457060
position (bp)

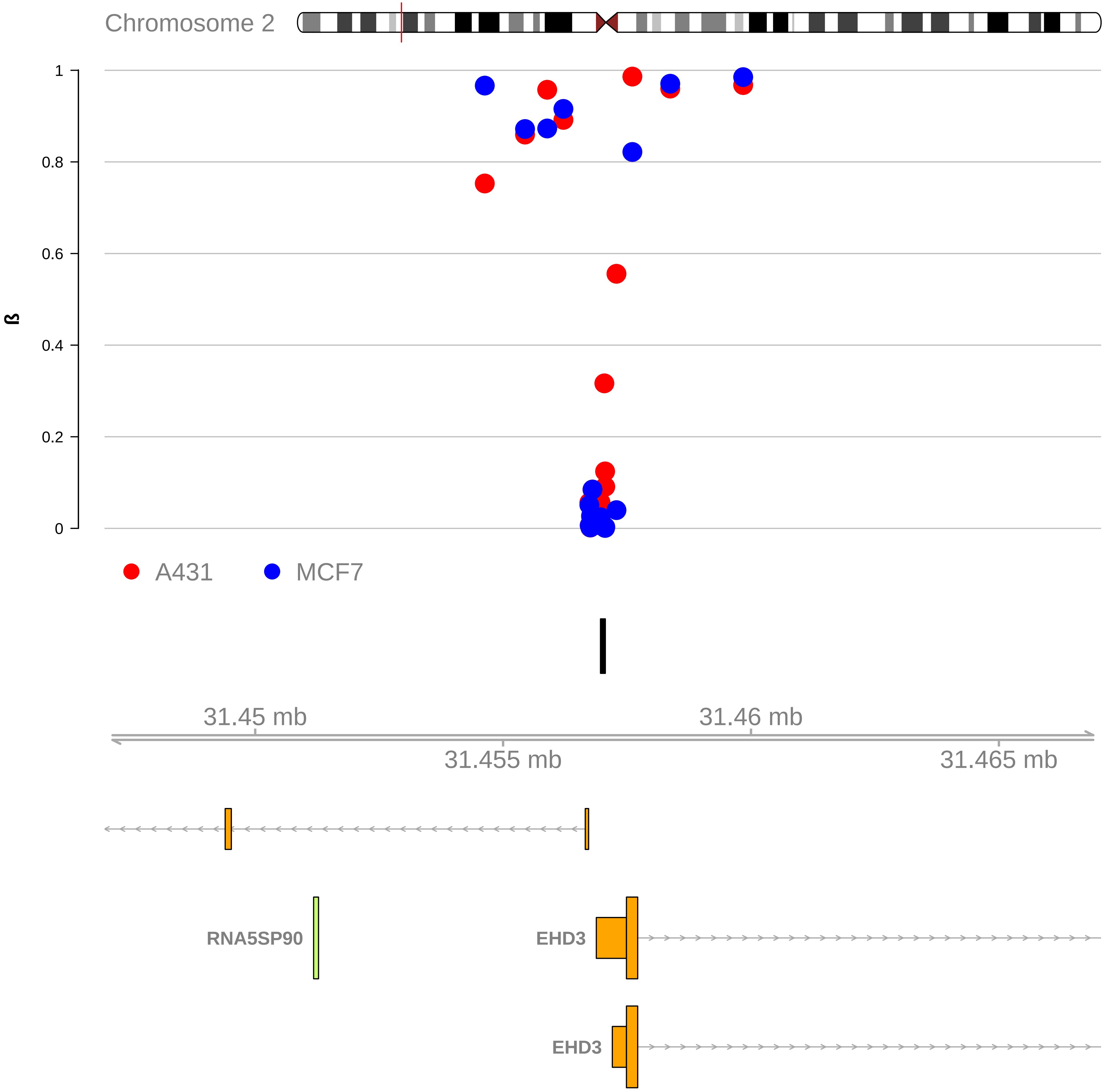

Chromosome 2
1
0.8
0.6
0.4
0.2
0
ß
A431
MCF7
31.45 mb
31.46 mb
31.455 mb
31.465 mb
RNA5SP90
EHD3
EHD3
